# Evaluation of Chemical Protocols for Inactivating SARS-CoV-2 Infectious Samples

**DOI:** 10.3390/v12060624

**Published:** 2020-06-08

**Authors:** Boris Pastorino, Franck Touret, Magali Gilles, Lea Luciani, Xavier de Lamballerie, Remi N. Charrel

**Affiliations:** Unité des Virus Émergents (UVE: Aix-Marseille Univ-IRD 190-Inserm 1207-IHU Méditerranée Infection), 13005 Marseille, France; boris.pastorino@univ-amu.fr (B.P.); franck.touret@univ-amu.fr (F.T.); magali.gilles@univ-amu.fr (M.G.); lea.luciani@ap-hm.fr (L.L.); xavier.de-lamballerie@univ-amu.fr (X.d.L.)

**Keywords:** SARS-CoV-2, coronavirus, extraction buffer, COVID-19, inactivation, lysis buffer

## Abstract

Clinical samples collected in coronavirus disease 19 (COVID-19), patients are commonly manipulated in biosafety level 2 laboratories for molecular diagnostic purposes. Here, we tested French norm NF-EN-14476+A2 derived from European standard EN-14885 to assess the risk of manipulating infectious viruses prior to RNA extraction. SARS-CoV-2 cell-culture supernatant and nasopharyngeal samples (virus-spiked samples and clinical samples collected in COVID-19 patients) were used to measure the reduction of infectivity after 10 min contact with lysis buffer containing various detergents and chaotropic agents. A total of thirteen protocols were evaluated. Two commercially available formulations showed the ability to reduce infectivity by at least 6 log 10, whereas others proved less effective.

## 1. Introduction

Coronavirus disease 2019 (COVID-19), classified as a pandemic by the World Health Organization (WHO), is a severe acute respiratory syndrome (SARS) caused by the virus designated as SARS-CoV-2 [1]. Since December 2019, measures to reduce person-to-person transmission of COVID-19 have been implemented to attempt to control the outbreak. Tremendous diagnosis efforts are done by healthcare workers by handling infectious samples from patients, and they are thus heavily exposed to risk of infection [2,3,4]. Accordingly, the WHO introduced laboratory guidelines to mitigate this risk for diagnosis and research activities [5]. Nonetheless, laboratory workers processing clinical samples continue to be exposed to the infectious SARS-CoV-2 [6]. Direct SARS-CoV-2 diagnosis is based on RNA detection by RT-qPCR from nasopharyngeal- or throat-swab [7] samples commonly containing high viral loads [8,9,10]. Methods for nucleic acid extraction rely on buffers to obtain high-quality nucleic acids that are not primarily developed for the inactivation of infectious samples. Automated nucleic acid extraction is generally performed outside of biosafety cabinets in which only noninfectious samples should be loaded. To achieve this objective, a prior inactivation step under appropriate biosafety conditions is an absolute requirement. Previous studies addressed the ability of lysis buffers added to the samples in the initial step of nucleic acid extraction to act as inactivation agents of several pathogenic viruses (including coronaviruses). However, discrepant results observed with dissimilar protocols led to controversial conclusions [11,12,13]. In this study, we tested different protocols on the basis of three different lysis buffers on SARS-CoV-2 culture supernatant and infected nasopharyngeal samples (virus-spiked samples and clinical samples collected in COVID-19 patients) to assess virus inactivation prior to nucleic acid extraction.

## 2. Materials and Methods

### 2.1. Cell Line

Vero-E6 cells (ATCC#CRL-1586) were grown at 37 °C in 5% CO_2_ with 1% penicillin/streptomycin (PS; 5000 U.mL^-1^ and 5000 µg.mL^-1^; Life Technologies), and supplemented with 1% nonessential amino acids (Life Technologies, Courtaboeuf, France) in Minimal Essential Medium (Life Technologies) with 5% FBS.

### 2.2. Viruses

The human 2019 SARS-CoV-2 strain (Ref-SKU: 026V-03883) was isolated at Charite University (Berlin, Germany) and obtained from the European Virus Archive catalog (EVA-GLOBAL H2020 project) (https://www.european-virus-archive.com). Experiments were performed in BSL3 facilities.

### 2.3. SARS-CoV-2 qRT-PCR

Total nucleic acids were purified using a Qiacube HT and a Cador pathogen extraction kit (both from Qiagen, Courtaboeuf, France). Viral RNA was quantified by RT-qPCR (qRT-PCR EXPRESS One-Step Superscript™, ThermoFisher Scientific, Illkirch-Graffenstaden, France) (10 min, 50 °C (1 cycle); 2 min, 95 °C (1 cycle); and 40 cycles, 95 °C, 3 sec/60 °C, 30 sec) using serial dilutions of a T7-generated synthetic RNA standard. Primers and probe targeted the N gene (fw: GGCCGCAAATTGCACAAT; rev: CCAATGCGCGACATTCC; probe: FAM-CCCCCAGCGCTTCAGCGTTCT-BHQ1. The calculated limit of detection was 10 RNA copies per reaction. 

### 2.4. Lysis Buffers

Three lysis buffers produced by Qiagen (Hilden, Germany) were tested. The approximate composition of each buffer was provided by Qiagen (18–20). ATL (1%–10% sodium dodecyl sulfate [SDS]), VXL (30%–50% guanidine hydrochloride, 1%–10% t-octylphenoxypolyethoxyethanol (Triton X-100)), and AVL (50%–70% guanidinium thiocyanate). AVL was also supplemented with (i) 100% ethanol on one hand (ii) and 1% Triton X-100 on the other hand.

### 2.5. SARS-CoV-2 Titration

SARS-CoV-2 was first propagated and titrated on Vero-E6 cells. Virus stock was diluted to infect Vero-E6 cells at an MOI of 0.001. Cells were incubated at 37 °C for 24–48 h, after which the medium was changed, and incubation was continued for 24 h. Supernatant was collected, clarified by spinning at 1500× g for 10 min, supplemented with 25mM HEPES (Sigma), and aliquoted. Aliquots were stored at −80 °C before titration. Virus infectivity was measured using a 50% tissue-culture infectivity dose (TCID_50_). Briefly, when cells were at 90% confluence, six replicates were infected with 150 μL of tenfold serial dilutions of the virus sample and incubated for 4 days at 37 °C under 5% CO_2_. Cytopathic effect (CPE) was read using an inverted microscope, and infectivity was expressed as TCID_50_/_mL_ on the basis of the Karber formula [14]. For reproducibility, the viral load was adjusted to a final titer of 10^6^ TCID_50_/mL in (i) infected-cell supernatant and (ii) in spiked nasopharyngeal samples.

### 2.6. Inactivation of Cell-Culture Supernatant

French norm NF EN 14476+A2 derived from European standard EN 14885 was used [15]. As recommended in the norms, 3 g/L bovine serum albumin (BSA, interfering substance) was added to infected-cell supernatants before inactivation for simulating “dirty” conditions [14] (Table 1). Each sample was incubated in duplicate with the lysis buffer at room temperature for 10 min; then, the buffer was discarded via ultrafiltration with Vivaspin 500 columns (Sartorius, Göttingen, Germany) as described [16]. The column was washed with 500 µL PBS three times and eluted in 250 µL of PBS, from which 100 µL was inoculated onto a Vero-E6 monolayer (90% confluence). Controls consisted of (i) uninfected Vero-E6 cells, (ii) Vero-E6 cells inoculated with the tested lysis buffer (cytotoxicity), and (iii) Vero-E6 cells inoculated with SARS-CoV-2 only. Cells were incubated at 37 °C under 5% CO_2_ for 5 days. The read-out was the presence of cytopathic effect (CPE) together with SARS-CoV-2 RNA detection through RT-qPCR at Day 5. In the absence of CPE at day 5, supernatant was passaged with the same read-out 5 days later (Day 10).

### 2.7. Inactivation of Nasopharyngeal Samples

Prepandemic nasopharyngeal samples were collected, pooled, and spiked with cell-culture supernatant to result in a final concentration of 6 Log_10_ TCID_50_/_mL_. Three arms consisting of 6 samples each were tested by using ATL, VXL, and AVL buffers, respectively. Two home-made enriched formulations containing AVL buffer were not tested. The same procedure was also evaluated on 6 individual clinical samples with a mean Ct value of 18 and a viral load greater than 4 Log_10_ TCID_50_/_mL_.

## 3. Results

### 3.1. Inactivation of Cell-Culture Supernatant

VXL and ATL buffers were able to inactivate SARS-CoV-2 with viral loads as high as 10^6^ TCID_50_/mL (Table 2). Virus replication was neither observed (CPE) nor assessed by RNA detection using RT-qPCR (Table 2). In contrast, the AVL buffer (GITC 50%–70%) alone did not inactivate SARS-CoV-2-infected supernatants since the four replicates resulted in virus growth as assessed by CPE (Table 2). The AVL buffer supplemented with absolute ethanol or 1% Triton X-100 resulted only in partial inactivation. CPE was observed for one in two samples, and SARS-CoV-2 RNA detection was confirmed by RT-qPCR. There was no clear impact of the presence of interfering proteins (BSA 3 g/L) (Table 2).

### 3.2. Inactivation of Nasopharyngeal Samples

For spiked nasopharyngeal samples, ATL and VXL buffers were able to reduce infectivity by at least 6 log10, as no CPE was observed for all replicates (Table 3). In contrast, the AVL buffer proved less efficient, as CPE was detected in 5 of 6 samples (Table 3). For patient samples, similar results were obtained with total virus inactivation observed using ATL or VXL buffers (Table 3). In contrast, and in accordance with previous results, the AVL buffer led to partial viral inactivation with the presence of CPE detected in 4 out of 6 samples after treatment (Table 3).

## 4. Discussion

Despite the previous emergence of SARS and MERS CoV, there are few studies on the inactivation protocols aimed at mitigating the risk of exposure towards coronaviruses for medical and laboratory personnel.

Qiagen is a prominent actor in the field of nucleic acid (NA) purification. Most other manufacturers of NA purification kits use a lysis buffer similar to ATL, AVL, and VXL. The ability of AVL to inactivate pathogenic viruses was debated, but there are no data for ATL and VXL. A total of ten different protocols using AVL, ATL, and VXL alone, or in association with ethanol or Triton-X100 were studied on SARS-CoV-2 according to the French version of the European recommended procedure (NF EN 14476+A2) [15], as previously shown for other viruses such as Ebola virus or foot and mouth disease virus [11,13,17,18]. Our results are in line with data reported for the Zaire ebolavirus [13]. These showed that GITC alone (AVL buffer), or supplemented with absolute ethanol, could not guarantee the complete inactivation of SARS-Cov-2 in cell culture (10^6^ TCID50), in 10^6^ TCID50-spiked nasopharyngeal samples or in nasopharyngeal swabs collected in COVID-19 acutely infected patients (10^4^ TCID50). The latter were worrisome in light of the 4/6 replicates that were still infectious despite the relatively low viral load before AVL buffer treatment. Results observed with virus-spiked nasopharyngeal samples were comparable with those obtained with cell-culture supernatant supplemented with interfering substances. It was shown that clinical samples with Ct values > 33–34 (corresponding to <3.16 TCID50) were not able to infect Vero-E6 cells [19]. In contrast, a recent study indicated that samples displaying Ct values >24 were not infectious [10]. Our results contradict those from Bullard et al. [10], whereas they are in line with those of La Scola et al. [19]. The reason for this discrepancy resides in the fact that, in Bullard et al. [10], Ct values were determined on fresh samples, whereas infectivity was measured after the same samples were frozen and thawed. The latter is known to reduce infectivity and result in a likely bias between RNA copies and infectivity.

Our results strongly suggested that ATL or VXL should be preferred to AVL. Our findings corroborate and expand recent results with SARS-CoV-2 [20]. Among many other kits, lysis buffer NucliSENS EasyMAG (BioMerieux) contains guanidine thiocyanate and Triton-X100 (undefined proportions); it is difficult, therefore, to anticipate its inactivation properties. Another example is the NucleoSpin RNA (Macherey-Nagel) lysis buffer that contains only guanidinium thiocyanate. It is, therefore, most likely that results observed with AVL apply to this kit. The composition of lysis buffers commercialized by ROCHE in HighPure Viral Nucleic Acid (ref#11858874001) and HighPure Viral RNA (ref#11858882001) kits contain guanidinium chloride (30%–50%), Triton-X100 (10%–20% (ref#11858874001), guanidinium chloride (30%–50%), and Triton-X100 30–50% (ref#11858882001)). Although not tested experimentally, extrapolation from our experiment results indicated that the aforementioned Roche lysis buffer may readily inactivate clinical samples containing SARS-CoV-2.

Since clinical samples collected in COVID-19-suspect patients are commonly manipulated in BSL-2 laboratories, the results presented in this study should help to choose the best-suited protocol for inactivation in order to prevent the exposure of laboratory personnel in charge of direct and indirect detection of SARS-CoV-2 for diagnostic purposes.

## Figures and Tables

**Table 1:** Protocols tested for assessing inactivation using lysis buffers.

Lysis Buffer	Composition ^a^	Nucleic Acid Extraction Kit (Catalog #)	Interfering Substance/Added	Lysis Buffer/Sample	Temperature (°C)	Contact Time (min)
Buffer ATL	1%–10% SDS ^b^	QIAsymphony DSP Virus/Pathogen Kits (#937036) or QIAsymphony DSP DNA Mini Kit (#937236)	±BSA ^f^ (3 g/L)	1:1	20	10
Buffer VXL	30%–50% GuHCl ^c^ + 2.5%–10% Triton X-100 ^d^	QIAmp cador Pathogen Mini kit (#54104) or QIAmp 96 DNA QIAcube HT kit (#51331)	±BSA (3 g/L)	1:1	20	10
Buffer AVL	50%–70% GITC ^e^	QIAamp Viral RNA Minikit (#52904)	±BSA (3 g/L)	4:1	20	10
			±BSA (3 g/L) + 1 volume ethanol 100%	4:1	20	10
			±BSA (3 g/L) + 1% Triton X-100 ^g^	4:1	20	10

^a^ as provided by Qiagen (www.qiagen.com > resources); ^b^ SDS = sodium dodecyl sulfate, ^c^ GuHCl = guanidine hydrochloride, *^d^* vol/vol, ^e^ GITC = guanidinium thiocyanate, ^f^ BSA = bovine serum albumin, ^g^ final concentration (vol/vol).

**Table 2:** SARS CoV-2 cell-culture supernatant inactivation using lysis buffers.

	Cell-Culture Supernatant (10^6^ TCID_50_/Ct Value 13.7)									
Inactivation Protocol	ATL Buffer		VXL Buffer		AVL Buffer		AVL Buffer + 100% Ethanol		AVL Buffer + 1% Triton X-100	
	Without BSA	BSA (3 g/L)	Without BSA	BSA (3 g/L)	Without BSA	BSA (3 g/L)	Without BSA	BSA (3 g/L)	Without BSA	BSA (3 g/L)
**Titer reduction**	≥10^6^	≥10^6^	≥10^6^	≥10^6^	<10^6^	<10^6^	<10^6^	≥10^6^	<10^6^	<10^6^
**CPE**	0/2	0/2	0/2	0/2	2/2	2/2	1/2	0/2	1/2	1/2
**RT-qPCR ^a^**	>40	>40	>40	>40	27	29	28	>40	30	28

Ct, cycle threshold, CPE, cytopathic effect tested in duplicate; BSA, bovine serum albumin. ^a^, mean Ct value when both replicates were positive.

**Table 3:** Inactivation of SARS CoV-2 positive nasopharyngeal samples using three lysis buffers.

	SARS-CoV-2-Spiked Nasopharyngeal Samples (10^6^ TCID_50_/Ct Value 13.7)			SARS-CoV-2 Nasopharyngeal Samples (1.2 × 10^4^ TCID_50_/Ct Value 18) ^b^		
	ATL Buffer	VXL Buffer	AVL Buffer	ATL Buffer	VXL Buffer	AVL Buffer
**Titer reduction**	**≥10^6^**	**≥10^6^**	**<10^6^**	**≥10^4^**	**≥10^4^**	**<10^4^**
**CPE**	**0/6**	**0/6**	**5/6**	**0/6**	**0/6**	**4/6**
**RT-qPCR ^a^**	**>40**	**>40**	**28**	**>40**	**>40**	**31**

Ct, cycle threshold, CPE, cytopathic effect tested in duplicate; ^a^, mean Ct value of positive replicates; ^b^, mean Ct and titer values.
